# Evaluating the Effectiveness of a Family-Based Virtual Childhood Obesity Management Program Delivered During the COVID-19 Pandemic in Canada: Prospective Study

**DOI:** 10.2196/40431

**Published:** 2022-11-03

**Authors:** Kayla Nuss, Rebecca Coulter, Bianca DeSilva, Jeann Buenafe, Ronak Sheikhi, Patti-Jean Naylor, Sam Liu

**Affiliations:** 1 School of Exercise Science Physical and Health Education University of Victoria Victoria, BC Canada; 2 Childhood Obesity Foundation Vancouver, BC Canada

**Keywords:** childhood obesity management, virtual intervention, COVID-19 pandemic, COVID-19, children, healthy lifestyle, health promotion, virtual health, digital health intervention, parenting, obesity, childhood obesity

## Abstract

**Background:**

Generation Health (GH) is a 10-week family-based lifestyle program designed to promote a healthy lifestyle for families with children who are off the healthy weight trajectory in British Columbia, Canada. GH uses a blended delivery format that involves 10 weekly in-person sessions, and self-guided lessons and activities on a web portal. The blended program was adapted to be delivered virtually due to the COVID-19 pandemic. Currently, the effectiveness of the virtual GH program compared with that of the blended GH program remains unclear.

**Objective:**

We aimed to (1) compare the effectiveness of the virtual GH program delivered during the COVID-19 pandemic with that of the blended GH program delivered prior to the pandemic for changing child physical activity, sedentary and dietary behaviors, screen time, and parental support–related behaviors for child physical activity and healthy eating, and (2) explore virtual GH program engagement and satisfaction.

**Methods:**

This study used a single-arm pre-post design. The blended GH program (n=102) was delivered from January 2019 to February 2020, and the virtual GH program (n=90) was delivered during the COVID-19 pandemic from April 2020 to March 2021. Families with children aged 8-12 years and considered overweight or obese (BMI ≥85th percentile according to age and sex) were recruited. Participants completed preintervention and postintervention questionnaires to assess the children’s physical activity, dietary and sedentary behaviors, and screen time, and the parent’s support behaviors. Intervention feedback was obtained by interviews. Repeated measures ANOVA was used to evaluate the difference between the virtual and blended GH programs over time. Qualitative interviews were analyzed using thematic analyses.

**Results:**

Both the virtual and blended GH programs improved children’s moderate-to-vigorous physical activity (*F*_1,380_=18.37; *P*<.001; ηp^2^=0.07) and reduced screen time (*F*_1,380_=9.17; *P*=.003; ηp^2^=0.06). However, vegetable intake was significantly greater in the virtual GH group than in the blended GH group at the 10-week follow-up (*F*_1,380_=15.19; *P*<.001; ηp^2^=0.004). Parents in both groups showed significant improvements in support behaviors for children’s physical activity (*F*_1,380_=5.55; *P*=.02; ηp^2^=0.002) and healthy eating (*F*_1,380_=3.91; *P*<.001; ηp^2^=0.01), as well as self-regulation of parental support for children’s physical activity (*F*_1,380_=49.20; *P*<.001; ηp^2^=0.16) and healthy eating (*F*_1,380_=91.13; *P*<.001; ηp^2^=0.28). Families in both groups were satisfied with program delivery. There were no significant differences in attendance for the weekly in-person or group video chat sessions; however, portal usage was significantly greater in the virtual GH group (mean 50, SD 55.82 minutes) than in the blended GH group (mean 17, SD 15.3 minutes; *P*<.001).

**Conclusions:**

The study findings suggested that the virtual GH program was as effective as the blended program for improving child lifestyle behaviors and parental support–related behaviors. The virtual program has the potential to improve the flexibility and scalability of family-based childhood obesity management interventions.

## Introduction

Children who are off the healthy weight trajectory have increased risks of chronic diseases, psychological distress, and lower quality of life [1]. The prevalence of children who are overweight or obese (BMI ≥85th percentile according to age and sex) has increased from 23% in the late 1970s to 35% in 2004 in Canada [2]. A similar trend was seen in the United States, where the prevalence of obesity in children and youth tripled between the late 1970s and 2016 from 5% to 18.5% [3]. A recent study reported that the rate of BMI increase almost doubled during the COVID-19 pandemic compared to the prepandemic period among children aged 2 to 19 years [4]. Consequently, there is an urgent need to develop innovative solutions to help families with children who are off the healthy weight trajectory.

Physical inactivity, increased screen time, and unhealthy food choices have all contributed to overweight or obesity among children [1,5]. The lockdown imposed during the COVID-19 pandemic has further exacerbated these unhealthy lifestyle behaviors. Recent studies have shown that physical activity significantly reduced, while screen time significantly increased among Canadian children [6]. There was also a significant increase in the consumption of unhealthy foods and beverages, such as sugary drinks, among children during the COVID-19 pandemic [7]. Therefore, lifestyle interventions aimed at promoting physical activity and a healthy diet, and reducing screen time are desperately needed for families with children who are off the healthy weight trajectory.

Family-based lifestyle interventions have been shown to be effective for managing childhood obesity [8-11]. Family-based interventions encourage the whole family to make lifestyle behavior changes and remove the focus from the child with overweight or obesity. Engagement with the entire family is important to improve a child’s lifestyle behaviors, since family-level attitudes and behaviors play critical roles in shaping a child’s lifestyle behaviors [11]. Based on the evidence supporting family-based interventions in combatting childhood obesity, our team collaborated with stakeholders, the Childhood Obesity Foundation, and the British Columbia Ministry of Health to develop a 10-week early intervention program, which was rebranded as “Generation Health” (GH) for families with children (8-12 years of age) who were off the healthy weight trajectory (BMI ≥85th percentile according to age and sex). Childhood obesity management interventions for children aged 8 to 12 years can be particularly effective as prepubertal children are more likely to return to a normal course of growth [12,13]. GH was designed to meet the needs of families living in British Columbia, Canada. GH used a blended in-person and online delivery model to provide program delivery flexibility for families. In our previous trial, this program was shown to be effective relative to a control in improving a child’s days of moderate-to-vigorous physical activity (MVPA), and parental support behaviors and self-regulation support for child physical activity and healthy eating [14]. Unfortunately, physical distancing restrictions and the temporary closure of recreation centers as a result of the COVID-19 pandemic did not allow in-person GH component delivery in March 2020. Consequently, our team rapidly adapted GH to be delivered completely virtually starting in April 2020. The overall curriculum of the virtual GH program remained the same as the blended GH program. However, the 10 weekly in-person sessions were adapted to be delivered using online group video sessions, and the online portal was updated to incorporate additional COVID-19–related interactive content (eg, video and audio lessons). The effectiveness of the virtual GH program delivered during the COVID-19 pandemic has not been previously evaluated. Thus, the study objectives were (1) to compare the effectiveness of the virtual GH program delivered during the COVID-19 pandemic with that of the blended GH program delivered prior to the COVID-19 pandemic for changing children’s physical activity, sedentary behaviors, dietary behaviors, and screen time, and parental support–related behaviors for child physical activity and healthy eating; and (2) to explore virtual GH program engagement and satisfaction. We hypothesized that (1) the virtual GH program would be as effective as the blended GH program in improving a child’s lifestyle behaviors and parental support–related behaviors and (2) families in the virtual GH program would have similar engagement and program satisfaction as those in the blended GH program.

## Methods

### Study Design

This study used a single-arm pre-post comparison design. Eligible families participated in study assessments at baseline and following the 10-week intervention. Families were invited for an exit interview at the end of the study to collect qualitative program feedback data. The blended GH program was delivered and evaluated from January 2019 to February 2020. The virtual GH program was delivered and evaluated during the COVID-19 pandemic from April 2020 to March 2021. All participants enrolled in the blended and virtual GH programs were included in this analysis. Families were recruited using social media; email mailouts to provincial networks; and posters displayed in recreation centers, medical offices, and schools.

### Ethics Approval

This study was approved by the research ethics board at the University of Victoria (H20-00564).

### Participants

Families with at least one child between the ages of 8 and 12 years and considered overweight or obese (BMI ≥85th percentile according to age and sex) were included. At least one parent/caregiver was required to participate in the program. Children with one or more comorbidities were excluded and referred to the Shapedown British Columbia clinical program.

### Program

#### Blended GH

The blended GH program was delivered at the following local community centers in British Columbia, Canada: Prince George (YMCA of Northern British Columbia), Kelowna (YMCA of Okanagan), Surrey (Tong Louie Family YMCA), Surrey (City of Surrey), Burnaby (City of Burnaby), and Greater Victoria (West Shore Parks and Recreation Society). The program was theoretically informed by the multi-process action control (M-PAC) framework, which emphasizes social cognitive approaches to facilitate intention formation, adoption of action control through self-regulation, and an action control maintenance phase where behavior becomes habitual and self-identified [15]. The in-person component consisted of 10 weekly 120-minute group sessions delivered by trained facilitators at local community centers and community-based activities (eg, family grocery store tour led by a registered dietitian). The weekly in-person sessions included specific child activities (eg, physical activity games developing basic physical literacy skills such as throwing, kicking, and catching), parent activities (eg, facilitator-led discussion about using behavior change techniques as tools for modifying families’ dietary or physical activity behaviors, reducing screen time, and developing parental support behaviors for child dietary and physical activity behaviors), and family activities (eg, family goal setting, physical activities, and recipes). The online component consisted of self-guided lessons for healthy living, which included a variety of physical activities, healthy eating activities, positive mental health family activities, and additional resources for parents. The online component complemented the weekly in-person group sessions. These online resources could be accessed via a mobile-friendly web portal. See Multimedia Appendix 1 for session activities and intervention details.

#### Virtual GH

The virtual GH program contained the same curriculum and used the same theoretical framework (M-PAC) as the blended GH program; however, the content of the program was adapted to be delivered online over a group video call (Zoom, Zoom Video Communications). Family activities were modified to accommodate this new delivery format. Program modifications included (1) reformatting the layout of each session (eg, front-loading all family time, replacing child-only physical activity time with family physical activity time, and ending with parent-only discussion time); (2) modifying activities and games for at-home delivery; and (3) replacing the additional community-based activities with virtual expert sessions (eg, virtual cooking classes with a registered dietitian, and virtual question and answer sessions with a physical activity or mental health expert). The self-guided component of the online portal was enhanced to include additional interactive videos and content to help families achieve a healthy lifestyle during the COVID-19 pandemic lockdown (eg, in-home fun family activities, screen time management tips, and resources for parents to support child dietary and physical activity behaviors). See Multimedia Appendix 1 for session activities and intervention details.

### Procedure

Study data were collected from the parents and children using an online questionnaire at baseline and at follow-up. Demographic data, including the child’s age and ethnicity, parents’ education, annual household income, number of people in the household, and family structure status (ie, single parent), were collected at baseline. Child BMI was collected by a research assistant at the delivery sites for the blended program. However, child BMI was self-reported by parents for the virtual program owing to physical distancing measures.

### Child Measures

#### Children’s MVPA

The Physical Activity Questionnaire for older children (PAQ-C) was used to evaluate the number of days in the past week that children engaged in 60 minutes of MVPA [16]. Specifically, the question stated, “During the past week (7 days), on how many days were you physically active for a total of at least 60 minutes per day? Count all the time you spent doing activities that increased your heart rate or made you breathe hard.” The response options were 0 to 7 days. The PAQ-C has been previously validated to assess MVPA among Canadian children and has a moderate correlation to the objective measures of MVPA (r=0.34, 95% CI 0.29-0.39) [17].

#### Children’s Screen Time and Sedentary Behaviors

The Physician-based Assessment & Counseling for Exercise (PACE) adolescent psychosocial instrument was used to measure screen time and sedentary behavior [18]. The validity of the questionnaire has been previously demonstrated (ρ=0.4) [19-21]. The questionnaire assessed the number of hours on a school day and a weekend day that children engaged in sedentary behaviors (ie, sitting on the couch) and screen time behaviors (ie, using a smartphone, television, iPad, or computer). The responses ranged from 0 hours to 6 or more hours.

#### Children’s Dietary Behaviors

Child dietary behaviors (ie, fruit and vegetable intake and sugary beverage intake) were assessed using questions drawn from the Centre for Disease Control and Prevention Behavioral Risk Factor Surveillance System (BRFSS) 7-day recall (intraclass correlation=0.50) [22]. The BRFSS survey included a 7-day recall with questions, such as, “in the last 7 days, how many times did you eat a green leafy or lettuce salad, with or without other vegetables?” and “in the last 7 days, how many times did you eat doughnuts, brownies, pies, or cakes?” The responses represented the number of times in the past week that the child consumed the items (1: none, 2: 1-3 times, 3: 4-6 times, 4: 1 time per day, 5: 2 times per day, 6: 3 time per day, and 7: 4 or more times per day).

### Parental Support Behaviors

#### Parental Support for Healthy Eating and Physical Activity

A subscale drawn from the Family Life, Activity, Sun, Health, and Eating (FLASHE)-EAT survey (α=.77) [23] and the Parent Physical Activity Support survey (α=.72) [24,25] were used. The FLASHE-EAT survey and healthy eating items (5-point Likert Scale; 1, strongly disagree to 5, strongly agree) were “I have to make sure that my child eats enough fruits and vegetables,” “I encourage my child to try different kinds of fruits and vegetables,” and “Bought fruit or vegetables you know your child likes.” The parental support for physical activity items (5-point Likert Scale; 1, strongly disagree to 5, strongly agree) were “I go out of my way to enroll my child in sports and other activities that get him/her to be physically active (eg after school programs and programs at the YMCA),” “I often watch my child participate in sporting activities (eg, watch your child perform at a softball game or dance recital),” and “I take my child to places where he/she can be active.”

#### Self-regulation for Parental Support of Child Healthy Eating and Physical Activity

The Parent Support of Child Physical Activity Questionnaire was adapted from previous research [25-27] for measuring self-regulation for eating (α=.86) and physical activity (α=.89). This subscale assessed parents’ regulation of their children’s physical activity and healthy eating behaviors by measuring parents’ goals and plans to support their children’s behaviors over the next month. Specifically, the items were “I set short-term (daily or weekly) goals for how I could support my child’s healthy eating/leisure-time physical activity behaviors last month” and “If I did not reach my goal/one of my goals for supporting my child’s healthy eating/physical activity last month, I analyzed what went wrong,” “I made plans regarding what to do if something made it difficult to support my child’s healthy eating/physical activity last month,” and “I made regular plans concerning when, where, how, and what kind of support I could provide for my child’s eating behaviors and food choices/physical activity last month.”

### GH Engagement

Weekly GH program attendance for the in-person and virtual group video sessions was recorded by facilitators using a tracking form. Web analytics captured the total minutes spent interacting with the web portal content. The average minutes per week a family spent logged into the portal was calculated by dividing the total time by 10 (the length in weeks of the GH program).

### Program Satisfaction

Program feedback questionnaires for participants were administered at the end of the interventions. The surveys prompted participants to (1) rate the weekly sessions (eg, please select whether you “liked” this session on a scale of 1 [“not at all”] to 5 [“a lot”]), (2) rate the level of satisfaction with intervention components (ie, family classroom, child physical activity, parent classroom, online portal, etc), and (3) rate the information given in weekly sessions (ie, was the information given in weekly sessions easy to understand, culturally suitable for your family, etc, with answers on a 5-point Likert scale ranging from 1 [“not at all”] to 5 [“a lot”]). Parents were also invited for a phone interview to provide further program feedback.

### Statistical Analysis

All statistical analyses were conducted using R statistical software version 4.1.0 (R Foundation for Statistical Computing). We determined that the data were missing at random and performed mean imputation for missing outcome variables [28]. Independent samples *t* tests and chi-square tests were conducted to compare continuous and categorical demographic variables between groups, respectively. A repeated measures ANOVA was conducted to examine the main effects of time (baseline and follow-up), as well as the group (blended GH vs virtual GH) by time (baseline and follow-up) interaction for all outcome variables. Independent *t* tests were used to evaluate program satisfaction and engagement for the blended and virtual GH programs. All quantitative statistical techniques used in this study to generate the results had established a significance set at *P*<.05. Qualitative data from postprogram interviews on program feedback were transcribed using Transcriptive software (Digital Anarchy, Inc) and analyzed using NVivo 12 (QSR International). General categories and themes were identified using a framework analysis approach [29]. Themes were then summarized into areas of program improvements.

## Results

### Participant Characteristics

Overall, 192 participants were enrolled in the GH program and completed baseline surveys. Participants’ demographic data are shown in Table 1. There was no significant difference between the blended and virtual GH groups in terms of children’s age and ethnicity, household income, and the number of single parents. The mean child age was 10.10 (SD 1.63) years, and 50.0% (96/192) of the children who attended the GH programs were female. The GH programs reached a demographic representing the British Columbia population [30], whereby 45.8% (88/192) of the children were white, 6.3% (12/192) were indigenous, 12.0% (23/192) were Asian (South Asian, West Asian, Chinese, and Southeast Asian), and 7.3% (14/192) were black or Latin American. Of the 192 participants, 102 (53.1%) were in the blended GH program and 90 (46.9%) were in the virtual GH program. Of the 102 participants in the blended GH program, 71 (69.6%) completed the program and provided follow-up responses. Meanwhile, of the 90 participants in the virtual GH program, 62 (68.9%) completed the program. Demographic characteristics of the completers and noncompleters of the GH programs are shown in Multimedia Appendix 2. We found that the percentage of completion was significantly higher for nonsingle parents than for single parents in both the blended and virtual GH programs (χ^2^_6_ [N=192]=18.03; *P*=.01).

**Table 1:** Demographic information of participants in the blended and virtual Generation Health programs.

Characteristic		Blended GH^a^ group (n=102)	Virtual GH group (n=90)	*P* value	
Child age (years), mean (SD)		10.24 (1.53)	9.82 (1.82)	.11	
Child BMI (>85th to ≤97th percentile), n (%)		41 (40)	41 (46)	.12	
Child BMI (>97th percentile), n (%)		61 (61)	49 (54)	.14	
Female child, n (%)		52 (51)	44 (49)	.97	
Adults in household, mean (SD)		2.19 (1.07)	1.89 (0.64)	.02	
Children in household, mean (SD)		1.94 (0.88)	1.94 (0.84)	.98	
**Child ethnicity, n (%)**					
	Indigenous	8 (7.8)	4 (4.4)	.23	
	White	43 (42.2)	45 (50.0)	.15	
	Asian (South Asian, West Asian, Chinese, and Southeast Asian)	15 (14.7)	8 (8.8)	.37	
	Black	6 (5.9)	2 (2.2)	.37	
	Latin American	2 (2.0)	4 (4.4)	.57	
	Arab	2 (2.0)	2 (2.0)	>.99	
	Other	17 (16.7)	12 (13.3)	.66	
	Missing values	8 (7.8)	7 (7.8)	>.99	
**Household income (CAD$^b^)**					
	<$28,000	9 (8.8)	8 (8.9)	>.99	
	$28,000 to <$34,000	5 (4.9)	1 (1.1)	.27	
	$34,000 to <$41,000	6 (5.9)	4 (4.4)	.90	
	$41,000 to <$47,000	5 (4.9)	6 (6.7)	.83	
	$47,000 to <$53,000	6 (5.9)	6 (6.7)	>.99	
	$53,000 to <$59,000	5 (4.9)	3 (8.9)	.86	
	≥$59,000	42 (41.2)	44 (48.9)	.35	
	Prefer not to answer	16 (15.7)	11 (13.9)	.63	
	Missing values	16 (7.8)	14 (7.8)	>.99	
**Single parent**					
	Yes	25 (24.5)	15 (16.7)	.34	
	No	64 (62.7)	66 (73.3)	.16	
	Prefer not to answer	5 (4.9)	2 (2.2)	.55	
	Missing values	8 (7.8)	7 (7.8)	>.99	

^a^GH: Generation Health.

^b^A currency exchange rate of CAD $1=US $0.73 is applicable.

### Children’s Physical Activity, Sedentary Behavior, Screen Time, and Dietary Outcomes

There was a main effect of time for days of MVPA and screen time (Table 2), suggesting that children in both groups reported significantly more days of reaching 60 minutes of MVPA (*F*_1,380_=18.37; *P*<.001; ηp^2^=0.07) and significantly lower screen time (*F*_1,380_=9.17; *P*=.003; ηp^2^=0.06 ). We also observed a significant interaction between group and time for vegetable intake among children. Specifically, participants in the virtual GH group reported significantly greater vegetable intake than those in the blended GH group at the 10-week follow-up (*F*_1,380_=15.19; *P*<.001; ηp^2^=0.004). No significant main effect of time or a group-by-time interaction was observed for fruit intake, sugary drink intake, or sedentary time (*P*>.05).

**Table 2:** Children’s dietary, physical activity, sedentary behavior, and screen time data before and after the blended and virtual Generation Health programs.

Variable	Blended GH^a^ group, mean (SD)			Virtual GH group, mean (SD)		Overall, mean (SD)			Main effect of time		Time-by-group interaction	
	Pre	Post	Pre		Post		Pre	Post		*P* value		*P* value
Fruit intake (times per day in a typical week)	3.20 (1.21)	3.27 (0.94)	2.92 (0.95)		3.11 (0.52)		3.07 (1.10)	3.20 (0.77)		.60		.51
Vegetable intake (times per day in a typical week)	2.55 (0.94)	2.44 (0.78)	2.25 (0.62)		2.73 (0.52)^b^		2.41 (0.82)	2.58 (0.68)		.28		<.001
Child’s sugary drink intake (times per day in a typical week)	1.72 (0.79)	1.60 (0.64)	1.70 (0.90)		1.41 (0.45)		1.71 (0.84)	1.51 (0.56)		.24		.22
60 min of MVPA^c^ (days per week)	3.46 (1.81)	4.34 (1.29)	3.31 (1.60)		4.03 (1.03)		3.39 (1.71)	4.20 (1.18)^d^		<.001		.59
Sedentary time (hours per day)	3.35 (1.44)	3.24 (1.05)	3.97 (1.23)		3.53 (0.78)		3.64 (1.21)	3.38 (0.94)		.37		.72
Screen time (hours per day)	3.50 (1.38)	3.01 (1.01)	3.85 (1.32)		3.12 (0.72)		3.66 (1.36)	3.06 (0.88)^d^		.003		.43

^a^GH: Generation Health.

^b^Significantly higher in the blended group after the 10-week intervention.

^c^MVPA: moderate-to-vigorous physical activity.

^d^Overall effect of time is significantly higher after the 10-week intervention.

### Parental Support Behaviors for Child Physical Activity and Dietary Behaviors

We detected a main effect of time for parental support for healthy eating (*F*_1,380_=3.91; *P*<.001; ηp^2^=0.01), self-regulation of support for healthy eating (*F*_1,380_=91.13; *P*<.001; ηp^2^=0.28), parental support for physical activity (*F*_1,380_=5.55; *P*=.02; ηp^2^=0.002), and self-regulation of support for physical activity (*F*_1,380_=49.20; *P*<.001; ηp^2^=0.16). After the intervention, parents reported higher scores on all these variables compared to the findings at baseline (Table 3). We also detected a significant group-by-time interaction for parental support for healthy eating (*F*_1,380_=3.91; *P*=.04; ηp^2^=0.01) and parental support for physical activity (*F*_1,380_=6.66; *P*=.01; ηp^2^=0.02). In both cases, parents in the blended GH group scored significantly higher than parents in the virtual GH group at follow-up.

**Table 3:** Preintervention and postintervention parental support for healthy eating and physical activity outcome variables.

Variable	Blended GH^a^ group, mean (SD)			Virtual GH group, mean (SD)			Overall, mean (SD)			Main effect of time		Time-by-group interaction
	Pre	Post	Pre		Post	Pre		Post	*P* value		*P* value	
Parental support for healthy eating	10.16 (1.17)	10.66 (0.79)^b^	10.09 (1.07)		10.21 (0.69)	10.13 (1.12)		10.46 (0.78)^c^	<.001		.04	
Self-regulation of support for healthy eating	11.63 (3.58)	15.25 (2.25)	11.27 (3.30)		14.83 (1.86)	11.46 (3.45)		15.05 (2.08)^c^	<.001		.39	
Parental support for physical activity	23.13 (3.62)	24.15 (2.67)^b^	22.81 (3.33)		22.20 (2.67)	22.98 (3.48)		23.24 (2.84)^c^	.02		.01	
Self-regulation of support for physical activity	12.42 (3.38)	14.99 (2.43)	12.17 (3.24)		14.56 (2.00)	12.30 (3.29)		14.79 (2.24)^c^	<.001		.26	

^a^GH: Generation Health.

^b^Significantly higher in the blended group after the 10-week intervention.

^c^Overall effect of time is significantly higher after the 10-week intervention.

### Program Attendance

Blended GH attendance at the weekly in-person sessions was 77% for those who completed the program. Similarly, virtual GH attendance at the weekly group sessions was 76% for those who completed the program. There was no significant difference between blended and virtual GH completion rates (*P*=.65). Web portal usage was significantly greater for the virtual GH program than the blended GH program. Families who completed the blended GH program spent an average of 17 (SD 15.3) minutes per week on the family portal, while families in the virtual GH program spent 50 (55.82) minutes (*P*<.001).

### Program Satisfaction

Overall, parents were highly satisfied with both the blended and virtual programs. Nearly all parents who completed satisfaction surveys indicated that the weekly program sessions helped them learn and were useful for changing their lifestyle. There was no significant difference in the mean program satisfaction score between the blended (3.9/5) and virtual (3.8/5) GH programs. Postprogram interviews with parents identified areas of improvement for the virtual GH program. These included (1) a reminder from delivery staff about upcoming sessions on the day of the program; (2) support for implementing lifestyle changes for families who do not have a nuclear family structure; (3) a more in-depth explanation of how to navigate the family portal; (4) additional cooking class sessions; and (5) additional resources to support goal setting after the program ends.

## Discussion

This study compared the effectiveness of a virtual GH program delivered during the COVID-19 pandemic with that of a blended GH program delivered prior to the pandemic. We observed that the virtual GH program was as effective as the blended GH program in improving child MVPA and reducing screen time. The virtual GH program appeared more effective than the blended GH program in improving vegetable intake among children. Additionally, parents in both the virtual and blended GH programs showed significant improvements in support behaviors for child physical activity and healthy eating, as well as self-regulation of support for child physical activity and healthy eating. Families in both the virtual and blended GH programs were satisfied with the program delivery. Overall, the findings from this study suggested that the virtual GH program was a feasible and effective option that has the added potential to improve the flexibility and scalability of delivering family-based childhood obesity management interventions.

Our results showed a large increase in child MVPA and a reduction in screen time following GH. Similar to the blended GH program, the virtual GH program added almost 1 day per week of at least 60 minutes of MVPA and reduced about 45 minutes of screen time per day. The multiple physical activity opportunities (eg, games and fundamental movement skills) during each session for children, the parent portal resources about limiting screen time and support for child physical activity, and the weekly family-based challenges may have contributed to intervention success. Our findings are consistent with the findings of previous studies. For example, a previous 12-week family-based childhood obesity management intervention (children aged 8-12 years) showed that MVPA increased by 53 minutes per week and screen time decreased by 34 minutes per day [31]. Similarly, in a previous 10-week family-based intervention (MEND) delivered in British Columbia, children showed an increase in weekly physical activity levels by 2.6 hours per week and a decrease in screen time by 3 hours per week following the intervention [10].

Furthermore, the findings about the levels of program engagement and satisfaction between the virtual and blended GH programs were noteworthy, as they suggested that families were willing to engage with the virtual delivery format. However, our results suggested that being a single parent may influence program completion, which has been previously reported [32,33]. Future studies must explore the potential reasons for not completing the program among single parents to help further improve intervention design. The increased portal engagement time may be a consequence of the additional interactive video and audio content. Conversely, it could be a consequence of more time at home during lockdown with less distractions and travel time for various activities. In our previous study evaluating the dose-response relationship of the blended GH program, we showed that the online GH portal complemented the in-person GH sessions. Specifically, additional engagements with the portal were associated with greater improvements in child physical activity and parental support behaviors, habits, and identity for physical activity [34]. Future research is warranted to explore the dose-response relationship for the virtual GH program. Overall, the results from this study are encouraging, especially since several studies have shown that child physical activity decreased while screen time increased during the pandemic [6,7].

Child vegetable intake following the intervention was significantly higher in the virtual GH group than in the blended GH group. This may have been due to the lockdown, as parents may have more opportunities to influence children’s vegetable intake while they are at home every day [25]. However, previous childhood obesity interventions delivered in-person have reported significant improvements in dietary behaviors [10,11,35,36]. The lack of significant changes in the intake of fruits and sugary drinks may reflect a ceiling effect. Children at baseline were already consuming fruits about 5 times per day and were drinking sugary drinks 0 to 3 times per week. Furthermore, the unit (times per day in a typical week) of measure for changes in fruit and vegetable intake used in this study may not be as sensitive as other assessment tools (eg, servings of fruits and vegetables) to detect changes over the study period. Future studies may consider the use of other assessment tools that may be more sensitive to changes [37].

The findings of this study have several implications for family-based interventions aimed at promoting a healthy lifestyle for children who are overweight or obese. First, this is one of the first studies to demonstrate the effectiveness of adapting a blended family-based program to be delivered virtually during the COVID-19 pandemic for Canadians living in British Columbia. Second, this study showed that virtual family-based interventions could be as effective and engaging as a blended program to promote a healthy lifestyle among children. This suggests that a virtual approach is another GH program delivery option for families even after the pandemic. The virtual delivery format has the potential to improve the flexibility and scalability of family-based lifestyle programs designed for children who are overweight or obese. The results from this study add to the existing body of literature showing the effectiveness of virtual and online health interventions [31,34-36]. The family feedback received (eg, reminder sessions, portal tutorials, and maintenance programs) can help inform future virtual intervention designs.

We recognize that this study is not without limitations. First, the program evaluation was only up to 10 weeks. Thus, the long-term effects of virtual and blended GH programs remain unclear, and future research is warranted. Second, this study lacked a control group, which may introduce potential bias. Third, we did not control for potential secular effects (eg, season and weather), which may influence lifestyle behaviors. Future studies are warranted to explore the effects of these potential variables on intervention effectiveness. Fourth, even though all the child and parental measures have been validated, the self-report measures may introduce potential bias. Furthermore, some questions used to assess parental support for physical activity were not pertinent during the COVID-19 pandemic. For example, parents were asked to respond to the statement, “I go out of my way to enroll my child in sports and other activities to get him/her to be physically active.” During the initial months of the COVID-19 pandemic, schools were closed and extracurricular activities for children were cancelled. Therefore, we cannot be sure that parent responses to these survey items accurately reflected their opinions and attitudes or the contextual factors. Finally, the children’s BMI was self-reported by caregivers during virtual GH delivery, and this may introduce bias. Future studies could consider collecting parental BMI, as it can influence a child’s weight and lifestyle behaviors [38]. Finally, this study used a pre-post comparison design, where data were collected during different time periods. Thus, caution is required when generalizing the results.

Overall, a 10-week family-based intervention (the GH program) was effective in improving days of MVPA among children and reducing screen time, regardless of the delivery method (blended vs virtual). Similarly, satisfaction was high across delivery methods. Our findings suggest that virtually delivered early intervention programs are not inferior to in-person programs and offer an alternative delivery approach that enhances program flexibility and potential scalability.

## Appendix

Multimedia Appendix 1 Blended Generation Health and virtual Generation Health program outline.

Multimedia Appendix 2 Demographic information of Generation Health program completers and noncompleters.

## Abbreviations

BRFSS: Behavioral Risk Factor Surveillance System
GH: Generation Health
FLASHE: Family Life, Activity, Sun, Health, and Eating
M-PAC: multi-process action control
MVPA: moderate-to-vigorous physical activity
PAQ-C: Physical Activity Questionnaire for older children

## References

[1] Tyson N, Frank M (2018). Childhood and adolescent obesity definitions as related to BMI, evaluation and management options. Best Pract Res Clin Obstet Gynaecol.

[2] Rao DP, Kropac E, Do MT, Roberts KC, Jayaraman GC (2016). Childhood overweight and obesity trends in Canada. Health Promot Chronic Dis Prev Can.

[3] Fryar C, Carroll M, Ogden C (2018). Prevalence of Overweight, Obesity, and Severe Obesity Among Children and Adolescents Aged 2–19 Years: United States, 1963–1965 Through 2015–2016. CDC.

[4] Lange SJ, Kompaniyets L, Freedman DS, Kraus EM, Porter R, Blanck HM, Goodman AB (2021). Longitudinal Trends in Body Mass Index Before and During the COVID-19 Pandemic Among Persons Aged 2-19 Years - United States, 2018-2020. MMWR Morb Mortal Wkly Rep.

[5] Liu S, Marques IG, Perdew MA, Strange K, Hartrick T, Weismiller J, Ball GDC, Mâsse LC, Rhodes R, Naylor P (2019). Family-based, healthy living intervention for children with overweight and obesity and their families: a 'real world' trial protocol using a randomised wait list control design. BMJ Open.

[6] Moore S, Faulkner G, Rhodes R, Brussoni M, Chulak-Bozzer T, Ferguson L, Mitra R, O'Reilly N, Spence J, Vanderloo L, Tremblay MS (2020). Impact of the COVID-19 virus outbreak on movement and play behaviours of Canadian children and youth: a national survey. Int J Behav Nutr Phys Act.

[7] Stavridou A, Kapsali E, Panagouli E, Thirios A, Polychronis K, Bacopoulou F, Psaltopoulou T, Tsolia M, Sergentanis TN, Tsitsika A (2021). Obesity in Children and Adolescents during COVID-19 Pandemic. Children (Basel).

[8] Kolotourou M, Radley D, Gammon C, Smith L, Chadwick P, Sacher PM (2015). Long-Term Outcomes following the MEND 7-13 Child Weight Management Program. Child Obes.

[9] Black AP, D'Onise K, McDermott R, Vally H, O'Dea K (2017). How effective are family-based and institutional nutrition interventions in improving children's diet and health? A systematic review. BMC Public Health.

[10] Liu S, Weismiller J, Strange K, Forster-Coull L, Bradbury J, Warshawski T, Naylor PJ (2020). Evaluation of the scale-up and implementation of mind, exercise, nutrition … do it! (MEND) in British Columbia: a hybrid trial type 3 evaluation. BMC Pediatr.

[11] Perdew M, Liu S, Naylor PJ (2021). Family-based nutrition interventions for obesity prevention among school-aged children: a systematic review. Transl Behav Med.

[12] Pandita A, Sharma D, Pandita D, Pawar S, Tariq M, Kaul A (2016). Childhood obesity: prevention is better than cure. Diabetes Metab Syndr Obes.

[13] Curbing Childhood Obesity: A Federal, Provincial and Territorial Framework for Action to Promote Healthy Weights. Government of Canada.

[14] Perdew M, Liu S, Rhodes R, Ball GD, Mâsse LC, Hartrick T, Strange K, Naylor P (2021). The Effectiveness of a Blended In-Person and Online Family-Based Childhood Obesity Management Program. Child Obes.

[15] Rhodes RE, Elliot AJ (2017). The Evolving Understanding of Physical Activity Behavior: A Multi-Process Action Control Approach. Advances in Motivation Science.

[16] Kowalski K, Crocker P, Donen R (2004). The Physical Activity Questionnaire for Older Children (PAQ-C) and Adolescents (PAQ-A) Manual. Pediatric Research in Sports Medicine.

[17] Marasso D, Lupo C, Collura S, Rainoldi A, Brustio P (2021). Subjective versus Objective Measure of Physical Activity: A Systematic Review and Meta-Analysis of the Convergent Validity of the Physical Activity Questionnaire for Children (PAQ-C). Int J Environ Res Public Health.

[18] Norman GJ, Vaughn AA, Roesch SC, Sallis JF, Calfas KJ, Patrick K (2004). Development of decisional balance and self-efficacy measures for adolescent sedentary behaviors. Psychology & Health.

[19] García-Cervantes L, Martinez-Gomez D, Rodriguez-Romo G, Cabanas-Sanchez V, Marcos A, Veiga OL (2014). Reliability and validity of an adapted version of the ALPHA environmental questionnaire on physical activity in Spanish youth. Nutr Hosp.

[20] Martínez-Gómez D, Martínez-De-Haro V, Del-Campo J, Zapatera B, Welk GJ, Villagra A, Marcos A, Veiga ÓL (2009). [Validity of four questionnaires to assess physical activity in Spanish adolescents]. Gac Sanit.

[21] Norris SL, Grothaus LC, Buchner DM, Pratt M (2000). Effectiveness of physician-based assessment and counseling for exercise in a staff model HMO. Prev Med.

[22] BRFSS Questionnaires. CDC.

[23] Mâsse LC, Lytle L (2017). Advancing Knowledge of Parent-Child Dyadic Relationships About Multiple Cancer Preventive Health Behaviors: The National Cancer Institute Family Life, Activity, Sun, Health, and Eating (FLASHE) Study. Am J Prev Med.

[24] Davison K, Li K, Baskin M, Cox T, Affuso O (2011). Measuring parental support for children's physical activity in white and African American parents: the Activity Support Scale for Multiple Groups (ACTS-MG). Prev Med.

[25] Rhodes RE, Spence JC, Berry T, Deshpande S, Faulkner G, Latimer-Cheung AE, O'Reilly N, Tremblay MS (2016). Understanding action control of parental support behavior for child physical activity. Health Psychol.

[26] Rhodes RE, Berry T, Faulkner G, Latimer-Cheung AE, O'Reilly N, Tremblay MS, Vanderloo L, Spence JC (2019). Application of the Multi-Process Action Control Framework to Understand Parental Support of Child and Youth Physical Activity, Sleep, and Screen Time Behaviours. Appl Psychol Health Well Being.

[27] Lithopoulos A, Liu S, Rhodes RE, Naylor P (2020). The role of identity in parental support for physical activity and healthy eating among overweight and obese children. Health Psychol Behav Med.

[28] Pigott TD (2010). A Review of Methods for Missing Data. Educational Research and Evaluation.

[29] Gale NK, Heath G, Cameron E, Rashid S, Redwood S (2013). Using the framework method for the analysis of qualitative data in multi-disciplinary health research. BMC Med Res Methodol.

[30] (2016). British Columbia Census Profile. Statistics Canada.

[31] Zoellner JM, Hill J, You W, Brock D, Frisard M, Alexander R, Silva F, Price B, Marshall R, Estabrooks PA (2017). The Influence of Parental Health Literacy Status on Reach, Attendance, Retention, and Outcomes in a Family-Based Childhood Obesity Treatment Program, Virginia, 2013-2015. Prev Chronic Dis.

[32] Williams N, Coday M, Somes G, Tylavsky F, Richey P, Hare M (2010). Risk factors for poor attendance in a family-based pediatric obesity intervention program for young children. J Dev Behav Pediatr.

[33] Davison K, Kitos N, Aftosmes-Tobio A, Ash T, Agaronov A, Sepulveda M, Haines J (2018). Corrigendum to "The forgotten parent: Fathers' representation in family interventions to prevent childhood obesity" [Prev. Med. 111 (2018) 170-176]. Prev Med.

[34] Liu S, Smith N, Nuss K, Perdew M, Adiputranto D, Naylor P (2022). Dose-Response Relationship of a Blended In-Person and Online Family-Based Childhood Obesity Management Program: Secondary Analysis of a Behavior Intervention. JMIR Pediatr Parent.

[35] Epstein L, Gordy C, Raynor H, Beddome M, Kilanowski C, Paluch R (2001). Increasing fruit and vegetable intake and decreasing fat and sugar intake in families at risk for childhood obesity. Obes Res.

[36] Taylor R, Cox A, Knight L, Brown D, Meredith-Jones K, Haszard J, Dawson A, Taylor B, Williams S (2015). A Tailored Family-Based Obesity Intervention: A Randomized Trial. Pediatrics.

[37] Naska A, Lagiou A, Lagiou P (2017). Dietary assessment methods in epidemiological research: current state of the art and future prospects. F1000Res.

[38] Fuemmeler BF, Lovelady CA, Zucker NL, Østbye T (2013). Parental obesity moderates the relationship between childhood appetitive traits and weight. Obesity (Silver Spring).

