# Supplementary material for: Evaluating the Effectiveness of a Family-Based Virtual Childhood Obesity Management Program Delivered During the COVID-19 Pandemic in Canada: Prospective Study
Source: JMIR Pediatr Parent. 2022 Nov 3;5(4):e40431. doi: 10.2196/40431 (PMC9635440; doi:10.2196/40431)
Supplement: Multimedia Appendix 1 [file pediatrics_v5i4e40431_app1.docx]

**Multimedia Appendix 1.** Blended Generation Health and virtual Generation Health program outline.

| **Weekly Session Title** | **Blended GH**  **Topics/activities** | **Virtual GH**  **Topics/activities** | **M-PAC construct target** | **Behavior Change Techniques** |
| --- | --- | --- | --- | --- |
| **Week 1:**  **Introduction to Generation Health** | **In-Person:**   - Introduction to program - Icebreaker game - Discuss Live 5-2-1-0+ (guidelines to encourage children and families to build healthy habits) - Children-specific and family physical activities and games - Tracking & Behavioral Change (Where Are You At Now? Healthy Living Goal Trackers) - Family activity: discover hidden sugar in common beverages - Overview division of responsibilities in feeding, Health for EveryBODY, Habit & Identity - Gratitude Circle   **Portal:**   - Parent resources about Parenting Practices that Promote Health - Parent resources on Promoting Healthy Attitudes About Body Size - Complete weekly family challenges and recipes | **Virtual:**   - Introduction to program - Icebreaker game - Discuss Live 5-2-1-0+ (guidelines to encourage children and families to build healthy habits) - Family physical activities and games – *modified to use props commonly found at home (participants asked to bring these)* - Tracking & Behavioral Change (Where Are You At Now? Healthy Living Goal Trackers) *– Zoom “breakout rooms” used to discuss individual and family goals* - Family activity: discover hidden sugar in common beverages *– created interactive slide deck* - Overview division of responsibilities in feeding, Health for EveryBODY, Habit & Identity - Gratitude Circle   **Portal:**   - Parent resources about Parenting Practices that Promote Health – *additional videos content adapted for the lockdown* - Parent resources on Promoting Healthy Attitudes About Body Size - Complete weekly family challenges and recipes | Initiating reflective processes (instrumental attitude, outcome expectations) | Information about health consequences (5.1)*, Behavioral practice/rehearsal (8.1)*, Self-monitoring (2.3)*, |
| 1. **Week 2:** 2. **Introduction to Active Living & Setting SMART Goals** | **In-Person:**   - Children-specific physical activities to promote fundamental movement skills through games; family games - Discuss the Benefits of Active Living and Physical Activity - Tops Tips to Become a Physically Active Family - Action Break: Chair Aerobics - Learn about S.M.A.R.T. Goal Setting - Healthy Living Goal Tracker – Children and Family - Review Family Portal   **Portal:**   - Practice SMART goal setting for physical activity - Parent resources about Top Tips on becoming active - Complete weekly family challenges and recipes | **Virtual:**   - Family physical activity to promote fundamental movement skills *– modified for a smaller space* - Discuss the Benefits of Active Living and Physical Activity - Tops Tips to Become a Physically Active Family *– families paired into breakout rooms to discuss* - Action Break: Chair Aerobics - Learn about S.M.A.R.T. Goal Setting– *use breakout rooms and whiteboard to facilitate discussion and increase interactivity* - Healthy Living Goal Tracker *– use “share screen” function to show participants an example* - Review Family Portal   **Portal:**   - Practice SMART goal setting for physical activity - Parent resources about Top Tips on becoming active- *additional videos content adapted for the pandemic* - Complete weekly family challenges and recipes | Regulation processes | Goal setting (1.1)*, Self-monitoring (2.3)*, Behavioral practice/rehearsal (8.1)*, Graded tasks (8.7)*, Information about emotional consequences (5.6)* |
| 1. **Week 3:** 2. **Your Guide to Healthy Food Choices** | **In-Person:**   - Children-specific and family physical activities and games - Gratitude Circle - Family Fun with Vegetables and Fruit - Canada's Food Guide and Discuss Healthy Food Choices - Tracking & Behavioral Change – Accomplishments & Challenges - Review Portal   **Portal:**   - Parent resources for choosing healthy protein foods - Parent resources on healthy eating on a budget - Parent resources on being a good role Model - Complete weekly family challenges and recipes | **Virtual:**   - Family indoor/home physical activity games – *modified to use props commonly found at home (participants asked to bring these)* - Gratitude Circle - Family Fun with Vegetables and Fruit *– created interactive slide deck (participants can draw/type on screen)* - Mystery Food Game *– participants asked to find a vegetable or fruit in their home and bring it to computer* - Canada's Food Guide and Discuss Healthy Food Choices - Tracking & Behavioral Change – Accomplishments & Challenges – *use breakout room and whiteboard functions to facilitate discussion and increase interactivity* - Review Portal   **Portal:**   - Parent resources for choosing healthy protein foods - Parent resources on healthy eating on a budget - Parent resources on being a good role Model - Complete weekly family challenges and recipes | Regulation processes | Goal setting (1.1)*, Self-monitoring (2.3)*, Behavioral practice/rehearsal (8.1)*,Graded tasks (8.7)*, Information about emotional consequences (5.6)* |
| **Week 4:**  **What’s on the Label? / Reducing Sugary Drinks** | **In-Person:**   - Children-specific and family physical activities and games - Gratitude Circle - Learn about reading food labels and sugary drinks - Family activities to identify sugar in drinks - Tracking Behavioral Change – Accomplishments & Challenges - Participate in a grocery store tour with a Registered Dietitian   **Portal:**   - Practice reading food labels - Complete weekly family challenges and recipes | **Virtual:**   - Family indoor/home physical activity games – *modified to use props commonly found at home (participants asked to bring these)* - Gratitude Circle - Learn about reading food labels and sugary drinks *– use whiteboard and share screen functions to increase engagement* - Family activities to identify sugar in drinks - Tracking & Behavioral Change – Accomplishments & Challenges – *use breakout room and whiteboard functions to facilitate discussion and increase interactivity* - Participate in virtual Q&A with a Registered Dietitian - Grocery store tour videos   **Portal:**   - Practice reading food labels - Complete weekly family challenges and recipes | Instrumental Attitude; Perceived Capability & Opportunity | Goal setting (1.1)*, Self-monitoring (2.3)*, Behavioral practice/rehearsal (8.1)*,Graded tasks (8.7)*, Information about emotional consequences (5.6)*, Social support (3.3)*; Restructuring the physical environment (12.1)*; Instruction on how to perform a behavior (2.1)* |
| 1. **Week 5:** 2. **Body Self-Compassion and Appreciation** | **In-Person:**   - Children-specific and family physical activities and games - Gratitude Circle - Learn about ways to fostering optimism & gratitude to support positive mental health - Body Self-Compassion and Appreciation discussion and reflection; Body Scan exercise - Tracking Behavioral Change – Accomplishments & Challenges - Positive mental health activity: Sending Kind Wishes - Review Portal   **Portal:**   - Parent resources on developing a Positive Body image - Parent resources on building family identity around healthy eating and physical activity - Complete weekly family challenges and recipes | **Virtual:**   - Family indoor/home physical activity games – *modified to use props commonly found at home (participants asked to bring these)* - Gratitude Circle - Learn about ways to fostering optimism & gratitude to support positive mental health - Body Self-Compassion and Appreciation discussion and reflection; Body Scan exercise - Tracking & Behavioral Change – Accomplishments & Challenges – *use breakout room and whiteboard functions to facilitate discussion and increase interactivity* - Positive mental health activity: Sending Kind Wishes - Review Portal   **Portal:**   - Parent resources on developing a Positive Body image - Parent resources on building family identity around healthy eating and physical activity - Complete weekly family challenges and recipes | Affective Judgment; Perceived Capability | Goal setting (1.1)*, Self-monitoring (2.3)*, Behavioral practice/rehearsal (8.1)*,Graded tasks (8.7)*, Information about emotional consequences (5.6)*, Social support (3.3)*; Restructuring the physical environment (12.1)*; Instruction on how to perform a behavior (2.1)* |
| **Week 6:**   1. **Creating a Positive Family Mealtime** | **In-Person:**   - Children-specific and family physical activities and games - Gratitude Circle - Discuss mindful eating and hunger scale; Mindful Eating activity - Review Portal - Discuss ways to create a positive family mealtime - Tracking Behavioral Change – Accomplishments & Challenges   **Portal:**   - Parent resources on get kids to the table - Parent resources on Mindful Eating - Complete weekly family challenges and recipes | **Virtual:**   - Family indoor/home physical activity games – *modified to use props commonly found at home (participants asked to bring these)* - Gratitude Circle - Discuss mindful eating and hunger scale; Mindful Eating activity *- participants asked to bring small pieces of fruit* - Review portal - Discuss ways to create a positive family mealtime *– use whiteboard and share screen functions* - Tracking Behavioral Change – Accomplishments & Challenges   **Portal:**   - Parent resources on get kids to the table - Parent resources on Mindful Eating - Complete weekly family challenges and recipes | Regulation processes | Goal setting (1.1)*, Self-monitoring (2.3)*, Behavioral practice/rehearsal (8.1)*,Graded tasks (8.7)*, Information about emotional consequences (5.6)*, Social support (3.3)*; Restructuring the physical environment (12.1)*; Instruction on how to perform a behavior (2.1)* |
| 1. **Week 7:**   **Your Family’s Eating Style** | **In-Person:**   - Children-specific and family physical activities and games - Gratitude Circle - Discuss strategies to get everyone involved in food shopping meal planning and preparation - Discuss ways to identify and overcome barriers to physical activity - Family activity to trying new foods together - Parent activity: Family Style & Eating Together - Tracking Behavioral Change – Accomplishments & Challenges - Review Portal   **Portal:**   - Parent resources on making family meals Happen - Parent resources on identifying physical activity barriers - Parent resources on outdoor family activities ideas - Complete weekly family challenges and recipes | **Virtual:**   - Family indoor/home physical activity games – *modified to use props commonly found at home (participants asked to bring these)* - Gratitude Circle - Discuss strategies to get everyone involved in food shopping meal planning and preparation - *additional videos content adapted for the lockdown* - Discuss ways to identify and overcome barriers to physical activity - Family activity to trying new foods together - Parent activity: Family Style & Eating Together *– use whiteboard function to facilitate participation* - Tracking Behavioral Change – Accomplishments & Challenges - Review Portal   **Portal:**   - Parent resources on making family meals Happen - Parent resources on identifying physical activity barriers - Parent resources on outdoor family activities ideas - Complete weekly family challenges and recipes | Regulation processes | Goal setting (1.1)*, Self-monitoring (2.3)*, Behavioral practice/rehearsal (8.1)*,Graded tasks (8.7)*, Information about emotional consequences (5.6)*, Social support (3.3)*; Restructuring the physical environment (12.1)*; Instruction on how to perform a behavior (2.1)* |
| **Week 8:**  **Positive Parenting, Sleep Hygiene and Brainiacs** | **In-Person:**   - Children-specific and family physical activities and games - Gratitude Circle - Discuss sleep hygiene and ways to increase restful sleep schedules/routines - Practice strategies to help calm the body when stressed/overwhelmed - How Can Live 5-2-1-0+ Support Better Sleep? - Tracking Behavioral Change – Accomplishments & Challenges - Review Portal - Parent activity: Celebrating Accomplishments & Overcoming Challenges   **Portal:**   - Parent resources on how to help your child get better sleep - Parent resources on supporting healthy sleep with 5-2-1-0+ - Parent resources on Tips for setting the mood for sleep - Complete weekly family challenges and recipes | **Virtual:**   - Family indoor/home physical activity games – *modified to use props commonly found at home (participants asked to bring these)* - Gratitude Circle - Discuss sleep hygiene and ways to increase restful sleep schedules/routines - Practice strategies to help calm the body when stressed/overwhelmed - How Can Live 5-2-1-0+ Support Better Sleep? - Tracking Behavioral Change – Accomplishments & Challenges - Review Portal - Parent activity: Celebrating Accomplishments & Overcoming Challenges   **Portal:**   - Parent resources on how to help your child get better sleep - Parent resources on supporting healthy sleep with 5-2-1-0+ - Parent resources on Tips for setting the mood for sleep - Complete weekly family challenges and recipes | Regulation processes | Goal setting (1.1)*, Self-monitoring (2.3)*, Behavioral practice/rehearsal (8.1)*,Graded tasks (8.7)*, Restructuring the physical environment (12.1)*; Instruction on how to perform a behavior (2.1)* |
| **Week 9:**  **Cooking and Playing Together** | **In-Person:**   - Ancient & Indigenous Games - Gratitude Circle - Tracking Behavioral Change – Accomplishments & Challenges - Discuss ways to build habits for healthy lifestyle; focus on positive changes - Parent activity: discuss tips for cooking with kids   **Portal:**   - Parent resources on developing food skills for children - Parent resources on cooking with plant-based protein - Complete weekly family challenges and recipes | **Virtual*:**   - Children specific activity: Ancient & Indigenous games *- adapted for indoors* - Gratitude Circle - Tracking Behavioral Change – Accomplishments & Challenges - Discuss ways to build habits for healthy lifestyle; focus on positive changes - Present individual participant certificates with accomplishments - Parent activity: discuss tips for cooking with kids - Discuss ways to build family identity for healthy living and maintenance strategies - Review Live 5-2-1-0+ framework and tips for going forward - Local wayfinding & Family Portal resources   **Portal:**   - Parent resources on developing food skills for children - Parent resources on cooking with plant-based protein - Complete weekly family challenges and recipes | Regulation processes, reflexive processes (habit) | Goal setting (1.1)*, Self-monitoring (2.3)*, Behavioral practice/rehearsal (8.1)*,Graded tasks (8.7)*, Social support (3.3)*; Instruction on how to perform a behavior (2.1)*, Habit formation (8.3)* |
| **Week 10:**  **Continuing Positive Change, Dance and Celebration** | **In-Person:**   - Children-specific and family physical activities and games - Gratitude Circle - Tracking Behavioral Change – Accomplishments & Challenges - Discuss ways to build family identity for healthy living and maintenance strategies - Review Live 5-2-1-0+ framework and tips for going forward - Local wayfinding & Family Portal resources - Present individual participant certificates with accomplishments   **Portal:**   - Parent resources on setting guidelines for active living, healthy eating, and sleep - Complete weekly family challenges and recipes | **Portal:**   - Parent resources on setting guidelines for active living, healthy eating, and sleep - Complete weekly family challenges and recipes | Regulation processes, reflexive processes (identity) | Goal setting (1.1)*, Self-monitoring (2.3)*, Behavioral practice/rehearsal (8.1)*,Graded tasks (8.7)*, Social support (3.3)*; Valued self-identity (13.4)* |

**Note: *Weeks 9/10 of Virtual GH combined (Cooking and Playing Together & Continuing Positive Change)**
