# Supplementary material for: Evaluating the Effectiveness of a Family-Based Virtual Childhood Obesity Management Program Delivered During the COVID-19 Pandemic in Canada: Prospective Study
Source: JMIR Pediatr Parent. 2022 Nov 3;5(4):e40431. doi: 10.2196/40431 (PMC9635440; doi:10.2196/40431)
Supplement: Multimedia Appendix 2 [file pediatrics_v5i4e40431_app2.docx]

**Multimedia Appendix 2.** Demographic information of Generation Health program completers and noncompleters.

|  | **Blended GH** | | **Virtual GH** | | **p-value** |
| --- | --- | --- | --- | --- | --- |
|  | ***Completed***  ***n=71*** | ***Incomplete***  ***n=31*** | ***Completed***  ***n=62*** | ***Incomplete***  ***n=28*** |  |
| **Child’s Age** | 10.04 [1.45] | 10.73 [1.69] | 9.57 [1.94]^a^ | 10.25 [1.53] | .04 |
| **Child BMI (> 85^th^ to ≤ 97^th^ percentile)** | 24(33.8%) | 16(51.6%) | 25 (40.3%) | 14(50.0%) | .27 |
| **Child BMI (> 97^th^ percentile)** | 47 (66.2%) | 15 (48.4%) | 37 (59.7%) | 14 (50.0%) |  |
| **Female child** | 36 (51%) | 16(53%) | 31(50%) | 13 (48%) | .53 |
| **Adults in Household** | 2.25 [0.94] | 2.04 [1.34] | 1.86 [0.47] | 1.96 [0.95] | .11 |
| **Children in Household** | 1.91 [0.90] | 2.00 [0.90] | 1.94 [0.76] | 1.92 [1.02] | .97 |
| **Child Ethnicity** |  |  |  |  |  |
| Indigenous | 3(4.5%) | 5(18.5%) | 2 (3.6%) | 2(8.7%) | .19 |
| White | 34(51.5%) | 9(33.3%) | 32 (57.1%) | 13(56.5%) |  |
| Asian (South Asian, West Asian, Chinese and South East Asian) | 13(19.70%) | 4(14.81%) | 10 (17.9%) | 4(17.4%) |  |
| Black | 2(3.0%) | 4(14.8%) | 1(1.8%) | 1(4.3%) |  |
| Latin American | 2(3.0%) | n/a | 1(1.8%) | n/a |  |
| Arab | n/a | n/a | 1(1.8%) | n/a |  |
| Other | 12(18.2%) | 5(18.5%) | 9 (16.1%) | 3 (13.0%) |  |
| **Household Income** |  |  |  |  |  |
| Less than $28,000 | 6(9.0%) | 3(11.1%) | 3 (5.1%) | 5(20.8%) | 0.63 |
| $28,000 to less than $34,000 | 2(3.0%) | 3(11.1%) | 1 (1.7%) | n/a |  |
| $34,000 to less than $41,000 | 4(6.0%) | 2(7.4%) | 2(3.4%) | 2(8.3%) |  |
| $41,000 to less than $47,000 | 3(4.5%) | 2(7.4%) | 4(6.8%) | 2(8.3%) |  |
| $47,000 to less than $53,000 | 3(4.5%) | 3(11.1%) | 4(6.8%) | 2(8.3%) |  |
| $53,000 to less than $59,000 | 4(6.0%) | 1(3.7%) | 2(3.4%) | 1(4.2%) |  |
| $59,000 or more | 34(50.7%) | 8(29.6%) | 34(57.6%) | 10(41.7%) |  |
| Prefer Not To Answer | 11(16.4%) | 5(18.5%) | 9(15.3%) | 2(8.3%) |  |
| **Single Parent** |  |  |  |  |  |
| Yes | 12(17.9%) | 13(48.1%)^b^ | 7(11.9%) | 8(33.3%)^c^ | .01 |
| No | 51(76.1%) | 13(48.1%) | 51(86.4%) | 15(62.5%) |  |
| Prefer Not To Answer | 4(6.0%) | 1(3.7%) | 1(1.7%) | 1(4.2%) |  |

Note: mean [SD]; n(%); ^a^Bonferroni post-hoc Virtual GH completed < Blended GH Incomplete, p<.05; ^b^Bonferroni post-hoc blended GH incompleted < blended GH completed p<.05; ^c^ Bonferroni post-hoc blended GH incompleted < blended GH completed, p<.05
